# Case Series: Abscopal Benefit of Surgery in 3 Immunotherapy-Treated Patients With Unresectable Cancer

**DOI:** 10.1177/2324709618786319

**Published:** 2018-07-06

**Authors:** Bryan Oronsky, Chris Larson, Tony R. Reid, Corey A. Carter

**Affiliations:** 1 EpicentRx, San Diego, CA, USA; 2 University of California San Diego, La Jolla, CA, USA

**Keywords:** cancer, surgery, immunotherapy, unresectable disease

## Abstract

For all of the optimism that immunotherapy has engendered, the flip side is that 7/10 patients with susceptible tumor types do not respond, while in nonsusceptible tumor types the response rates are significantly lower. In contradiction of the current orthodoxy against surgery in the setting of unresectable disease, we present 3 examples of immunotherapy-treated patients with widespread recurrence who experienced dramatic clinical improvement following debulking/metastasectomy. Taken together with examples from the literature that correlate longer survival with surgical intervention during treatment with immunotherapy, these 3 cases suggest that a new paradigm involving a wider role for surgery in the management of these patients should be explored. Possible mechanisms by which surgery may synergize with immunotherapy and improve outcomes are also discussed.

## Introduction

Frequently lost in the hype and hope of immunotherapy is the reality that the majority of patients treated with PD-1 and CTLA-4 checkpoint inhibitors do not benefit. Response rates vary between 20% and 30% for tractable tumor types,^1^ that is, melanoma, lung, and renal cancers, which means that the rest of the patient population, 70% to 80%, is classified as nonresponders and accordingly fare worse, while in breast, pancreatic, microsatellite stable colorectal and esophageal cancers immunotherapy has been largely ineffective.^1,2^ The reasons for immune compromise and ineffective responses are multiple and include immunosuppressive cytokine production (eg, transforming growth factor-β, interleukin-10, vascular endothelial growth factor, and prostaglandin E2),^3^ the upregulation of immunoinhibitory immune checkpoint receptors on effector T-cells and myeloid cells, which induces a state of anergy or exhaustion, recruitment, and infiltration of immunosuppressive cells such as Tregs and myeloid-derived suppressor cells,^4^ decreased neoantigen burden with downregulation of MHC genes, and suppression of pro-inflammatory cytokine secretion.^5^ Selected tumor escape mechanisms that compromise the antitumor response are summarized in Table 1.

**Table 1.** Selected Tumor Escape Mechanisms.

Mechanism	Basis of Escape Mechanism
Ignorance	Lack of danger signals
	Decreased neoantigen burden
Impaired antigen presentation	Mutation or downregulation of tumor antigens
	Mutation or downregulation of MHC genes
Expression of immunosuppressive molecules	Cytokines (TGF-β, IL-10, VEGF, prostaglandins, etc)
	Checkpoint proteins (PD-1, CTLA-4, TIM-3, LAG-3, etc)
	Indolamine 2,3-diooxygenase (IDO)
Tolerance induction	Regulatory T cells and MDSCs
	Mitigation of pro-inflammatory cytokine secretion

Abbreviations: TGF-β, transforming growth factor-β; VEGF, IL-10, interleukin-10; vascular endothelial growth factor; MDSCs, myeloid-derived suppressor cells.

Current strategies to address these escape mechanisms, reverse immune tolerance, and break through the 30% checkpoint inhibitor response rate ceiling as well as to improve response rates in initial nonresponders include the adjunction of radiation, targeted biologics such as CAR-T cells and oncolytic viruses, cancer vaccines, and chemotherapy. Typically, noncurative surgery is not a strategy advocated in the metastatic/unresectable setting because in accordance with the central tenet of medicine “first do no harm” risk factors for increased morbidity and mortality including older age, poor performance status, and surgical complexity are generally present. Moreover, while early surgical excision results in long-term “cures,” a well-accepted premise, which has been discussed in the literature for over a century, is that later excision may stimulate the growth rate of metastases.^6^

**Figure 1.**
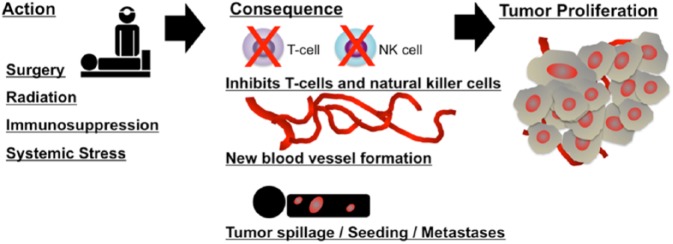
Surgery and other stimuli may affect angiogenesis and immune regulation, which in combination with other local microenvironment factors (eg, premetastatic niche cells) promote escape from tumor dormancy leading to tumor cell proliferation (green). The relationship of dormant tumor cells to cancer stem cells remains to be elucidated. Adapted from Tseng et al.^11^

Multiple lines of evidence have demonstrated the existence of a complex crosstalk between the primary tumor and metastatic foci such that in common with radiation therapy tumor surgical resection may actually result in a significant acceleration of the metastatic process.^6^ This effect, which appears to increase with primary tumor size, is potentially correlated with multiple mechanisms^7,8^ (Figure 1):

1. The production of various growth factors and proangiogenic factors in healing wounds, such as vascular endothelial growth factor, transforming growth factor-β, and basic fibroblast growth factor^9,10^
2. Removal of source of antiangiogenic factors, such as angiostatin and thrombospondin-1, secreted by the primary tumor^11^
3. Surgically induced suppression of cell-mediated immunity, particularly natural killer cell responses, which is directly related to the amount of surgical trauma and tissue damage^12^
4. Diffuse tumor spillage with lymphatic or hematogenous spread^13^

Several counterarguments, in favor of surgical intervention, include the following^14^:

1. Improved chemotherapy sensitivity of residual tumor
2. Less immunosuppressive factor release
3. Reduction of tumor stem cells
4. Spillage of tumor antigen in the circulation and presentation to the immune system with the activation/production of cytotoxic T-cells and antibodies

These counterarguments were in part culled from a literature search, which revealed exceptions to the general rule that surgery should be avoided in the unresectable metastatic setting unless the intent is purely palliative.^15,16^ To cite examples particularly relevant to this educational review, nephrectomy is commonly performed for patients with metastatic renal cell carcinoma based on improved survival with nephrectomy followed by interferon-α versus interferon-α alone,^17-22^ and in patients with metastatic melanoma who underwent complete metastasectomies after high-dose interleukin-2, overall survival was increased compared with historical data.^23^

Likewise, the experiences of the 3 late-stage immunotherapy-treated patients presented in this article who underwent metastasectomy/debulking are at odds with current clinical practice, which is supported by a large body of literature that advocates against the use of surgical intervention in precisely this setting. We have termed the observed paradoxical synergy between immunotherapy and surgery the “Surgical Abscopal Effect” as opposed to the radiotherapy one. While surgery is generally considered to be immunosuppressive, as described above, the release of tumor neoantigens combined with the reduction of tumor stem cells and the expression of pro-inflammatory cytokines in the presence of targeted immunotherapy may have been sufficient in these cases to shift the balance from T-cell tolerance to T-cell activation.

## Cases

### Case 1

Patient 1 is an 88-year-old white male, a retired pathologist, with a dual diagnosis of melanoma and squamous cell carcinoma of the left ear, neck, and forehead. A small flat patch had been observed since about 5 years. First diagnosed with a small retro-auricular melanotic growth, it grew rapidly and later examination revealed a large fungating mass that was warty, bulky, and elevated in appearance protruding from the left external auditory canal with involvement of the postauricular region and the mastoid area. A positron emission tomography scan demonstrated local spread to cervical lymph nodes without evidence of metastases.

**Figure 2.**
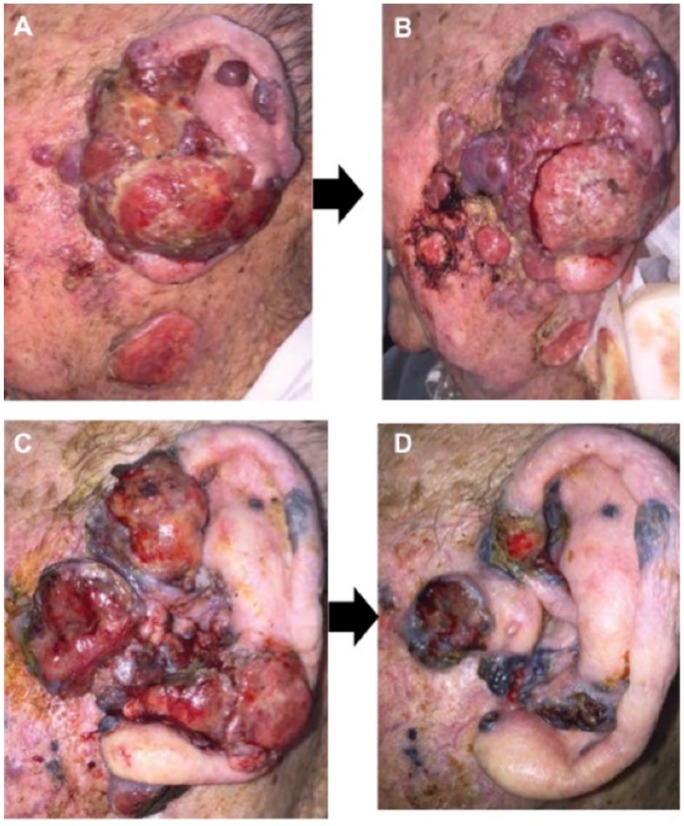
Patient lesion: progression over time. (A) Lesion prior to treatment in November. (B) Lesion post third infusion of nivolumab in December. (C) Lesion post surgery in February. (D) Lesion post ninth infusion of nivolumab in March.

Having been deemed an inappropriate candidate for curative resection due to the size and spread of the primary lesion, the patient was started on 3 mg/kg of the anti-PD-1 inhibitor, nivolumab, administered every other week, which appeared to result in rapid exophytic spread with increased production of blood-tinted (serosanguinous) discharge. A hypothesis of pseudoprogression recommended continuation of nivolumab. At patient’s insistence, aggressive resection/surgical debulking was performed with nivolumab continued perioperatively. Over the next few weeks, treatment with nivolumab resulted in significant shrinkage of the residual tumor, as shown in Figure 2.

### Case 2

Patient 2 is a 65-year-old white male with melanoma metastatic to the lungs, spine, abdomen, and coccyx. Prior treatment summary included resection of abdominal masses to relieve bowel obstruction, radiation to coccyx, and wedge resection of lung metastases in August 2014, since they were limited in number. Subsequently, he received 4 cycles of the anti-CTLA-4 inhibitor, ipilimumab, with a diagnosis of stable disease. Four months later, he was started on the anti-PD-1 inhibitor, nivolumab. Ten months later, he underwent debulking surgery of enlarging abdominal masses, diagnosed as inoperable, and resection of the coccyx metastasis, respectively. In January 2016, during repair of an abdominal wall defect (with nivolumab continued perioperatively), it was discovered that the tumors disappeared. Positron emission tomography/computed tomography scan demonstrated complete resolution of the abdominal masses and mild residual metabolic activity within the surgical cavity of the coccygeal mass, likely indicative of postsurgical/inflammatory change.

### Case 3

Patient 3 is a 47-year-old white female with squamous cell cervical cancer that originally presented as FIGO (Federation of International of Gynecologists and Obstetrician) stage 1B and was treated with radiation therapy. She subsequently developed recurrence with metastases in the lungs, adrenal gland, and paraspinal tissues and was treated with carboplatin/paclitaxel and bevacizumab and palliative radiotherapy (2700 cGy) to the paraspinal mass. On progression, she was started on a Phase I clinical trial called PRIMETIME (NCT02518958), which involves dosing of nivolumab with the experimental epigenetic and macrophage and cancer stem cell-targeting agent, RRx-001.

**Figure 3.**
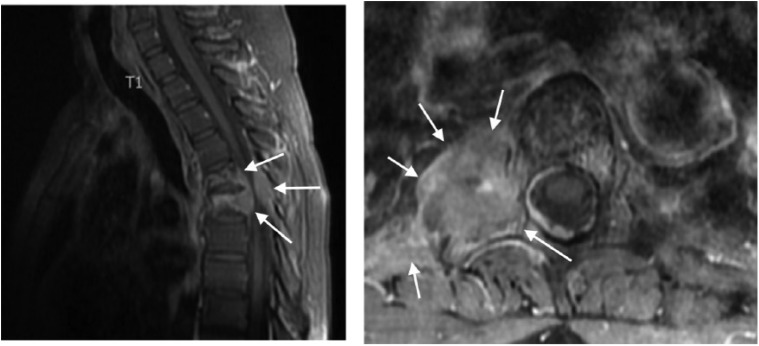
Invasion of the tumor at the level of T5.

At her first 6-week restaging scan, the patient showed stable disease with an approximately 10% reduction in tumor size. Her second 12-week restaging scan demonstrated significant growth of the paraspinal thoracic mass with apparent encroachment of the spinal canal at the level of T5 even while the rest of her lesions continued to diminish in size (Figure 3). However, most unusually, the patient did not describe any neurological symptoms. In fact, the day before, the patient went for a 3-mile run. Her chief—and only—complaint was back pain for which she took gabapentin (100 mg, PO) and oxycodone (5 mg, PO, PRN). On physical examination, the patient was neurologically intact with normal reflexes, muscle tone, and sphincter functions and negative Babinski signs.

One week later, the patient underwent surgical resection without incident and with postoperative resolution of her back pain. Pathology of the tumor showed the replacement of necrotic tumor cells with collagenous scar. The rest of her lesions continued to diminish in size in the absence of any treatment, possibly due to surgically induced immunogenicity effects.

## Conclusion

The term abscopal effect from the Latin *ab scopus*, meaning “away from the target,” describes the systemic bystander effects on nontargeted lesions with local radiotherapy due to the release of immunogenic tumor neoantigens and inflammatory factors such as tumor necrosis factor-α, which induces an enhanced immune response against unirradiated, malignant cells expressing similar tumor antigens.^24-26^ These out-of-field, action-at-a-distance effects have been previously described in multiple malignancies, including melanoma, lymphoma, and lung metastases of hepatocellular carcinoma.^27^ Despite its association with radiotherapy, abscopal effects have also been observed with hyperthermia and surgery.^28-30^

**Figure 4.**
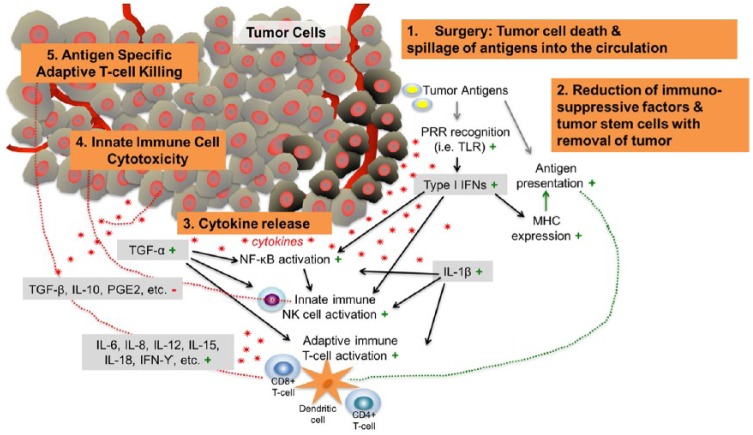
Proposed depiction of abscopal events that contribute to antitumor efficacy post surgery in combination with immunotherapy. In addition to tumor cell death, surgical tumor removal or debulking likely induces secretion of cytokines and chemokines that can kill cancer cells directly and also recruit and activate innate and adaptive immune cells that attack the tumor. Most of the downstream effects of surgery are favorable for tumor therapy (indicated by the green plus signs), which counteract immunosuppressive molecules (red minus sign) in the tumor microenvironment.

Accordingly, we hypothesize that abscopal responses resulting from the synergy between surgery and immunotherapy were responsible for the dramatic clinical benefits observed in these 3 patients (see Figure 4).

Also, immunotherapy was continued in the perioperative period in 2 of these 3 patients on the premise that it would help drive pro-inflammatory cytokine and chemokine release.

While further clinical correlation is necessary, repeated observations of this abscopal effect may drive a paradigm shift in the management of metastatic patients in which noncurative surgical treatment is routinely considered for its abscopal properties. The 3 cases presented in this educational review, who otherwise almost certainly would not have responded, may have benefited from the addition of this surgical option to the treatment armamentarium.
